# Beyond Exosomes: Biological Properties, Isolation Challenges, and Functional Redefinition of Non-Vesicular Extracellular Nanoparticles (NVEPs)

**DOI:** 10.3390/biom16091330

**Published:** 2026-09-13

**Authors:** Hengxiang Liu, Zishuai Chen, Hai He, Jiaxue Yuan, Xiaoyong Chen, Heqiang Yang, Sijing Yang, Zifan Wang, Zhiqiang Li

**Affiliations:** 1 Key Laboratory of Biotechnology and Bioengineering of State Ethnic Affairs Commission, Biomedical Research Center, School of Bioengineering, Northwest Minzu University, Lanzhou 730030, China; y252531008@stu.xbmu.edu.cn (H.L.); czs2410188708@stu.xbmu.edu.cn (Z.C.); y242530471@stu.xbmu.edu.cn (H.H.); y252530996@stu.xbmu.edu.cn (J.Y.); y241930514@stu.xbmu.edu.cn (X.C.); y242530459@stu.xbmu.edu.cn (H.Y.); y220830413@stu.xbmu.edu.cn (S.Y.); 2 Gansu Animal Cell Technology Innovation Center, Northwest Minzu University, Lanzhou 730030, China; 3 Key Laboratory of Oral Diseases of Gansu Province, Key Laboratory of Stomatology of State Ethnic Affairs Commission, School of Stomatology, Northwest Minzu University, No. 1 Xibeixincun Baiyin Road, Chengguan District, Lanzhou 730030, China

**Keywords:** non-vesicular extracellular nanoparticles, exomere, supermere, non-vesicular extracellular RNA, isolation and purification, functional redefinition, liquid biopsy

## Abstract

Extracellular vesicles (EVs) have dominated research on intercellular communication for decades. However, the discovery of exomeres in 2018 revealed that conventional exosome preparations contain large quantities of long-overlooked non-membranous nanoparticles. Additional non-vesicular extracellular nanoparticles (NVEPs), including supermeres and SECmeres, have since been reported. Most of these particles are smaller than 50 nm in diameter, yet they carry protein and RNA cargo that was previously thought to be delivered exclusively by exosomes. Consequently, certain functions once attributed to exosomes now require reassignment. This review summarizes the classification, cargo composition, and structural features of NVEPs. It then compares the respective limitations of differential ultracentrifugation, density gradient centrifugation, size-exclusion chromatography, and flow field-flow fractionation for NVEP isolation, together with strategies for their combined use. Furthermore, the functional mechanisms that distinguish NVEPs from vesicular EVs are discussed at three levels: the extracellular stability of non-vesicular extracellular RNA (nv-RNA), cellular uptake pathways, and downstream effects. Current evidence indicates that NVEPs are enriched for disease-associated cargo in cancer and neurodegenerative diseases and show potential for liquid biopsy and drug delivery. Nevertheless, ambiguous definitional boundaries, the absence of reference materials, and insufficient in vivo evidence jointly hinder quantification and clinical translation in this field. Because these limitations arise largely from the absence of unified technical criteria, establishing internationally recognized standards for isolation and characterization is a prerequisite for the maturation of NVEP research.

## 1. Introduction

### 1.1. Evolution of Research on the Heterogeneity of Extracellular Particles (ECPs)

Extracellular vesicles (EVs) are nanoscale particles naturally released by nearly all eukaryotic and prokaryotic cells and have long been regarded as key mediators of intercellular communication [1,2]. Early studies focused mainly on EVs as a heterogeneous population of lipid bilayer-enclosed structures, including exosomes, microvesicles, and apoptotic bodies, which differ markedly in size, biogenesis, and functional properties [3,4]. With the development of high-resolution analytical techniques and single-particle detection platforms, it has become clear that the complexity and diversity of EVs far exceed earlier assumptions. Such heterogeneity provides a rich source of biomarkers for disease diagnosis, but it also poses a major obstacle to the precise dissection of EV biological functions and their clinical translation [5,6,7]. In recent years, the research scope has expanded from membrane-bound vesicles alone to a broader spectrum of extracellular particles (ECPs), encompassing lipoproteins, RNA-binding protein complexes, and the newly identified non-vesicular extracellular nanoparticles (NVEPs) [8,9]. These advances have shifted the field from a generic description of “vesicles” toward the refined classification and functional characterization of distinct ECP subpopulations. Distinguishing these subpopulations in physiological and pathological processes has therefore become necessary [10,11].

### 1.2. Proposal of the NVEP Concept and the Scientific Necessity of Moving “Beyond Exosomes”

**Figure 1:**
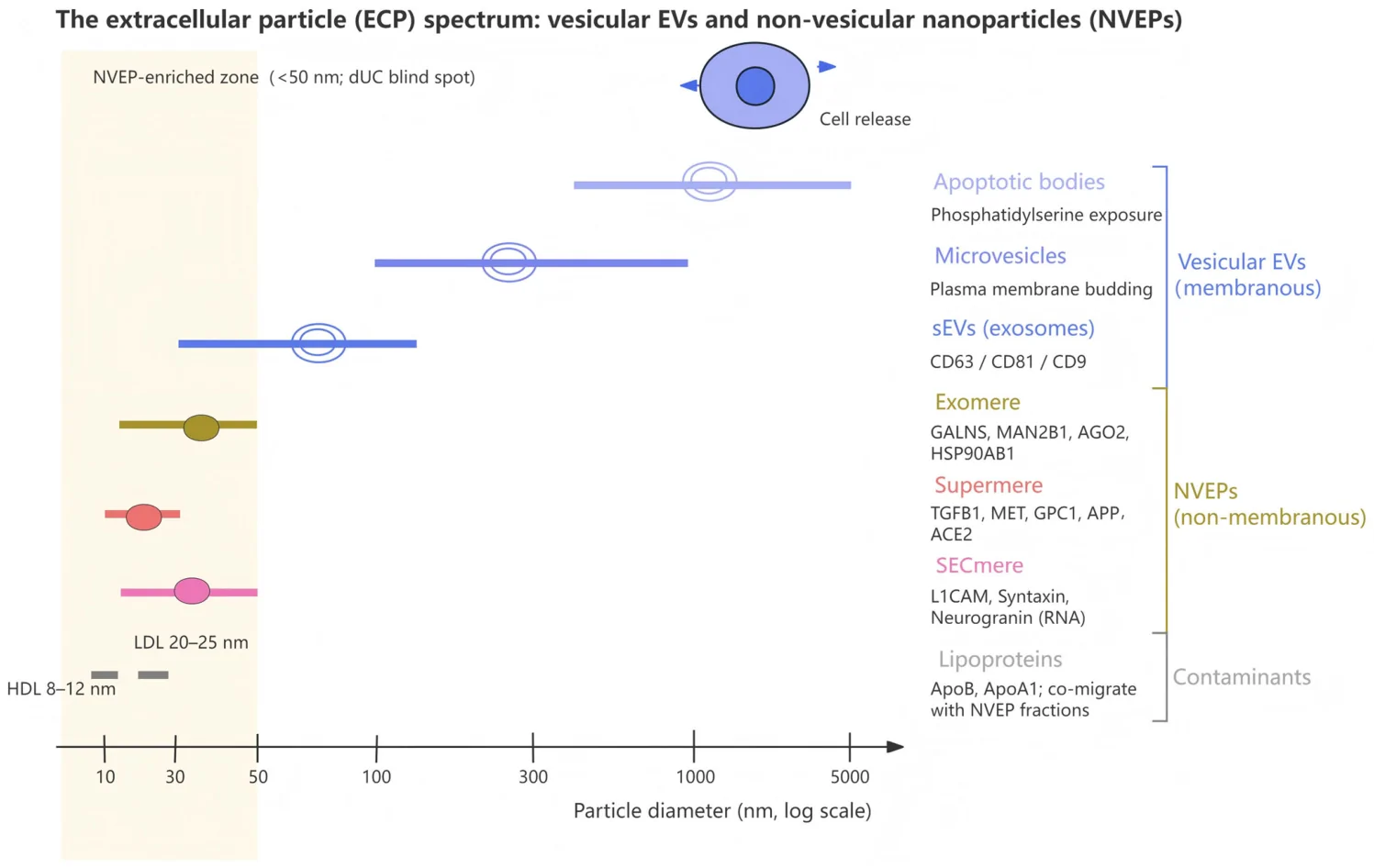
The extracellular particle (ECP) spectrum: classification of vesicular EVs and non-vesicular nanoparticles (NVEPs). The shaded area indicates the NVEP-enriched zone (<50 nm), i.e., the blind spot of conventional differential ultracentrifugation (dUC); representative markers are listed for each particle class.

Despite considerable progress in EV research, the recent discovery of non-membranous NVEPs, particularly exomeres and supermeres, has challenged the traditional definition of EVs, which centers on the lipid bilayer membrane [11,12]. Exomeres were first identified and named by Zhang et al. in 2018 using asymmetric flow field-flow fractionation (AF4) [13], revealing a non-vesicular component that had long been overlooked in conventional exosome preparations. The size ranges, membrane structural features, and representative markers of the vesicular and non-vesicular components within the extracellular particle spectrum are shown in Figure 1. Studies have shown that specific proteins and nucleic acids once widely believed to reside mainly in exosomes are in fact more enriched in these newly identified membrane-free NVEPs, prompting researchers to reassess the material basis of extracellular communication [12,14]. For example, supermeres have been found to carry cargo associated with cancer, Alzheimer’s disease (AD), and cardiovascular disease, and the majority of extracellular RNA is in fact associated with supermeres rather than with conventional small EVs [15]. In addition, novel NVEP subpopulations identified in the central nervous system, such as SECmeres, carry brain-specific markers and show greater diagnostic potential than small EVs in distinguishing patients with AD [16]. Establishing the NVEP concept therefore has dual significance: it refines the classification system of extracellular particles, and it corrects the possible misattribution of non-vesicular functions to exosomes in earlier studies, thereby enabling a more accurate dissection of the carriers of intercellular information transfer [9,11].

### 1.3. Confusion Between NVEPs and Conventional EVs and Its Impact on Functional Interpretation

The core problem currently facing this field is the co-isolation of NVEPs and conventional EVs during separation and purification, which means that many biological functions previously attributed to exosomes may have been misassigned. Owing to the lack of standardized isolation methods, conventional techniques such as differential ultracentrifugation often fail to effectively separate membrane-free NVEPs from membranous EVs, resulting in their admixture in the final preparations [8,14]. This confusion directly compromises the accuracy of functional interpretation. For example, certain functions reported to be mediated by exosomes, such as metabolic reprogramming and the transfer of drug resistance, may in fact be carried out by supermeres [15]. Moreover, although some non-vesicular extracellular RNA (nv-RNA) is vulnerable to nuclease degradation, it exhibits high stability within specific complexes and participates in intercellular communication, a mechanism fundamentally different from the vesicle-mediated protection proposed by traditional models [17,18]. In addition, NVEPs and EVs differ in their cellular uptake pathways and in vivo distribution [19,20]. Vesicular EVs have been shown to acquire a biomolecular corona that shapes their biological identity, cellular uptake, and in vivo fate [21,22,23,24]; whether membrane-free NVEPs acquire comparable coronas in biological fluids remains experimentally unresolved, as no study has yet directly profiled the corona composition or dynamics of exomeres or supermeres. Studying them without discrimination would severely impede the in-depth dissection of disease mechanisms and the development of precision therapeutic strategies. Therefore, clearly distinguishing NVEPs from EVs and critically re-evaluating previous findings have become an urgent requirement for improving the scientific rigor of this field [25].

### 1.4. Scope of This Review and Formulation of Key Scientific Questions

This review aims to systematically summarize the current research landscape of the emerging NVEP field, with a focus on the biological properties, isolation challenges, and functional redefinition of exomeres, supermeres, and other non-vesicular extracellular particles. We first describe the classification criteria, cargo composition, and structural features of NVEPs that distinguish them from conventional EVs. We then critically evaluate the limitations of existing isolation and purification techniques and discuss how to overcome the methodological bottlenecks caused by inadequate purity assessment and the lack of standardization. On this basis, we analyze the unique functional mechanisms of NVEPs and discuss the reassignment of functions previously attributed to exosomes. Subsequently, we summarize the empirical evidence for NVEPs in cancer, neurological disorders, and metabolic diseases, together with their translational prospects. Finally, this review distills the current theoretical controversies and unresolved questions, including the ambiguous boundary of the “non-vesicular” definition, the absence of gold-standard reference materials, and the discrepancies between in vitro and in vivo environments [8,11,18]. We further propose future perspectives on establishing internationally recognized characterization guidelines and a technical roadmap integrating multi-omics approaches, with the aim of clarifying the carrier composition of extracellular communication and advancing NVEP research toward a reproducible and quantifiable stage [25,26].

## 2. Classification, Cargo Composition, and Structural Features of NVEPs

### 2.1. Differences in Physicochemical Properties Between Exomeres and Supermeres: Size, Density, and Non-Membranous Structure

NVEPs represent a novel class of entities within the spectrum of ECPs that are distinct from conventional vesicles enclosed by a lipid bilayer, with exomeres and supermeres being the two most extensively characterized subtypes [12,27]. Unlike EVs, which typically range from 30 to 1000 nm in diameter and possess a well-defined lipid bilayer, exomeres and supermeres are defined as non-membranous nanoparticles that lack a typical phospholipid bilayer boundary [2,11,13]. In terms of physicochemical properties, these two NVEP subtypes display pronounced size heterogeneity. They are generally smaller than 50 nm in diameter and, in high-resolution (12–36% iodixanol) density gradients, are recovered in fractions distinct from those containing EVs, which makes them difficult to pellet effectively or readily confounded with soluble proteins in conventional differential ultracentrifugation protocols [14,16]. Density gradient centrifugation has further revealed their physical separation from EVs: whereas classical sEVs band at buoyant densities of approximately 1.13–1.19 g/mL in sucrose (approximately 1.08–1.13 g/mL in iodixanol), exomeres and supermeres partition into discrete zones of higher buoyant density, indicating that their internal assembly mechanisms and material composition differ fundamentally from those of vesicles [28]. Although morphologically distinct from exomeres, supermeres, as a newly identified subtype, show a markedly higher in vivo uptake rate than small extracellular vesicles (sEVs) and exomeres, suggesting that their unique surface physicochemical properties may mediate differential biological behaviors [15]. In addition, a class of small particles termed “SECmeres” has been identified in the human brain and blood. These particles likewise conform to the non-sEV criterion of <50 nm and are enriched in brain-specific markers, further confirming the dimensional and structural diversity of non-membranous nanoparticles and their tissue-specific distribution [16]. A comparison of the physicochemical properties of exomeres, supermeres, SECmeres, conventional sEVs, and common co-isolated contaminants such as lipoproteins is presented in Table 1. The clear delineation of these physicochemical properties constitutes the physical basis for separating NVEPs from conventional EV research and characterizing their functions independently. Systematic side-by-side isolation and multi-omics profiling of sEVs, exomeres, and supermeres have further delineated their distinct cargo profiles [29]. Representative protein markers for exomeres have been defined in previous work [30]. Of note, lipoproteins exhibit buoyant density profiles that partially overlap with high-density NVEP fractions [31,32]. The characterization of conventional sEVs in this review follows the MISEV2018 guidelines [33].

### 2.2. NVEP Cargo Spectrum: Proteins, Lipids, Metabolites, and nv-RNA

The cargo composition of NVEPs differs markedly from that of co-isolated EVs and exhibits a degree of subtype specificity. Regarding nucleic acid cargo, the majority of extracellular RNA is in fact associated with supermeres rather than with exosomes, as was long assumed. Although such nv-RNA is theoretically vulnerable to ribonuclease degradation, it displays relative stability in biological fluids and culture media, suggesting that it exists in the form of ribonucleoprotein complexes or other protective structures and participates in intercellular communication rather than merely in protein synthesis [15,17]. For example, exomeres have been shown to be enriched in RNA interference (RNAi) proteins, such as Argonaute-2 and HSP90AB1, together with specific microRNAs (miRNAs). These miRNAs remain highly resistant to nucleases even after protease and surfactant treatment, indicating that exomeres are highly stable non-vesicular complexes [18]. At the proteomic level, the protein profiles of EVs, exomeres, and supermeres remain consistently distinct regardless of culture conditions or isolation methods. Supermeres are particularly enriched in components involved in extracellular matrix (ECM) remodeling and in various disease-associated proteins, including cancer-associated glycolytic enzymes, TGFBI, MET, and GPC1, the AD-associated protein APP, and the cardiovascular disease-associated proteins ACE2, ACE, and PCSK9 [15,28,29]. With respect to lipids and metabolites, although culture conditions and purification strategies influence small RNA base modifications and lipidomic profiles, NVEPs, as non-membranous carriers, necessarily exhibit lipid composition patterns distinct from those of lipid bilayer-based EVs [29]. In addition, certain specific cargo, such as troponin, exists predominantly in a non-vesicular form in the plasma of patients with chronic heart failure, accounting for 40–60% of the total. This is in sharp contrast to healthy individuals, in whom troponin is mainly vesicle-associated, suggesting that the NVEP cargo spectrum may be dependent on pathological states. However, this observation is currently based on a single study and awaits validation in additional disease models [34]. Furthermore, antimicrobial small RNAs have been detected in the extracellular space of plant systems in a non-vesicular, protein-bound form and can enter bacterial cells to exert gene silencing [35], suggesting that non-vesicular cargo may be prevalent across species.

### 2.3. Biogenesis of NVEPs: Unresolved Questions and Indirect Clues

Compared with vesicular EVs such as exosomes, for which endosomal sorting complexes required for transport (ESCRT)-dependent and ESCRT-independent biogenesis pathways are relatively well defined, the biogenesis of NVEPs remains a “black box” in this field, with only scattered indirect clues available for inference. Because they lack a lipid bilayer, NVEPs cannot be generated through classical multivesicular body (MVB) fusion or plasma membrane budding. Their formation may instead involve the active secretion of macromolecular complexes or unconventional secretory pathways [11,36]. Several studies have pointed to potential regulatory nodes. For example, the ceramide transfer protein (CERT) interacts with phosphatidylinositol 4-phosphate (PI4P) on endosomes via its pleckstrin homology (PH) domain, mediating non-vesicular ceramide transport between the endoplasmic reticulum (ER) and MVBs and forming a complex with Tsg101 of the ESCRT-I machinery. Although this mechanism is mainly associated with exosome biogenesis, the “non-vesicular transport” elements it involves may provide molecular clues to the intracellular trafficking of NVEP components [37]. Under stress conditions, cells may activate alternative secretory programs. For instance, Plasmodium parasites release EVs enriched in aggregation-prone proteins via a Vps60-mediated pathway under heat shock stress to maintain proteostasis. Whether such stress-induced cargo redistribution also applies to the generation of membrane-free particles remains to be verified [38]. In addition, melanoma cells upregulate mitochondrial extrusion under oxidative stress as an alternative quality control mechanism, releasing free organelles that lack cristae. This phenomenon of non-vesicular organelle release suggests that cells may possess extensive non-vesicular material disposal pathways under specific stress conditions [39]. However, it must be acknowledged that no direct evidence currently elucidates the specific assembly machinery or secretory channels of exomeres and supermeres. Existing biogenesis models are largely based on analogies with EV pathways or on the exclusion of co-isolated contaminants. Dedicated tracing technologies are therefore urgently needed to clarify the biogenesis of NVEPs.

### 2.4. Molecular Fingerprints and Potential Identification Markers Revealed by Proteomics

Given that NVEPs lack universal membrane surface markers such as CD63 and CD81, proteomics has become a central tool for defining their identity, resolving their heterogeneity relative to EVs, and discovering specific identification markers. High-throughput mass spectrometry (MS) has successfully identified several NVEP-specific molecular fingerprints. N-acetylgalactosamine-6-sulfatase (GALNS) and mannosidase alpha class 2B member 1 (MAN2B1) have been confirmed as specific molecular markers of exomeres, enabling their effective discrimination from sEVs [30]. For supermeres, the protein fingerprint not only differs from those of sEVs and exomeres but also shows marked disease relevance. These particles are enriched in APP (AD), ACE2, ACE, and PCSK9 (cardiovascular diseases), and multiple cancer-associated proteins, making them potential targets for liquid biopsy [15]. In central nervous system (CNS) research, SECmeres have been found to be enriched in RNA transcripts of L1CAM, Syntaxin, and Neurogranin. Single-cell sequencing deconvolution further revealed their specific origin from the brain endothelial cells that constitute the blood–brain barrier (BBB). This is in sharp molecular contrast to sEVs, which are enriched in Synaptotagmin, α-synuclein, and MAPT and derive from a broad range of cellular sources [16]. In addition, studies on prostate cancer have indicated that, although precise classification remains challenging, protein profiling based on ECP characteristics has shown potential for cancer risk stratification [27]. The integration of database resources has further accelerated this process. Platforms such as Vesiclepedia and SVAtlas have incorporated proteomic data of non-vesicular particles, including exomeres and supermeres, providing standardized references for cross-study comparison and the mining of novel markers [40,41]. The NVEP molecular markers and characteristic cargoes reported to date are summarized in Table 2. These proteomic lines of evidence support the recognition of NVEPs as independent extracellular particle entities and provide a molecular basis for developing isolation and detection methods that do not rely on membrane markers.

## 3. Key Technical Bottlenecks: A Methodological Critique of NVEP Isolation and Purification

### 3.1. Loss and Co-Isolation of NVEPs During Differential Ultracentrifugation

Differential ultracentrifugation (dUC) has long been regarded as the gold standard for EV isolation. In NVEP research, however, this method has significant methodological blind spots. Because NVEPs such as exomeres and supermeres lack a lipid bilayer and differ in density from classical EVs, their behavior in standard dUC protocols is difficult to predict. Because of their small sedimentation coefficients, NVEPs remain suspended in the supernatants that are recovered and carried forward through the low-speed steps intended to remove cells, debris, and large vesicles, and they are ultimately discarded with the final supernatant whenever the terminal ultracentrifugation step fails to pellet them. Conversely, they readily co-precipitate nonspecifically with EVs, lipoproteins, or protein aggregates, resulting in severely inadequate purity of the isolates [14,42]. Studies have shown that samples prepared by dUC alone often contain large amounts of soluble immunosuppressive factors, such as vascular endothelial growth factor A (VEGF-A) and monocyte chemoattractant protein-1 (MCP-1). These contaminants can mask the true biological activity of NVEPs. For example, such preparations failed to induce the maturation of monocyte-derived dendritic cells (moDCs), an activity that became evident only in fractions purified by density gradient centrifugation combined with size-exclusion chromatography [42]. In addition, the high shear forces generated by dUC may cause structural damage to or aggregation of NVEPs, and its low throughput and time-consuming nature further limit its application in large-scale clinical sample analysis [43,44]. Therefore, although dUC remains suitable for the isolation of conventional sEVs, it is no longer suitable as the sole means of NVEP isolation, and its role in NVEP enrichment workflows needs to be reconsidered. It is generally recommended to use dUC as a preliminary concentration step rather than a terminal purification method, or to combine it with other high-resolution techniques to overcome its inherent limitations [45,46].

### 3.2. Comparison and Optimization of Density Gradient Centrifugation, SEC, AF4, and High-Resolution Fractionation Techniques

**Figure 2:**
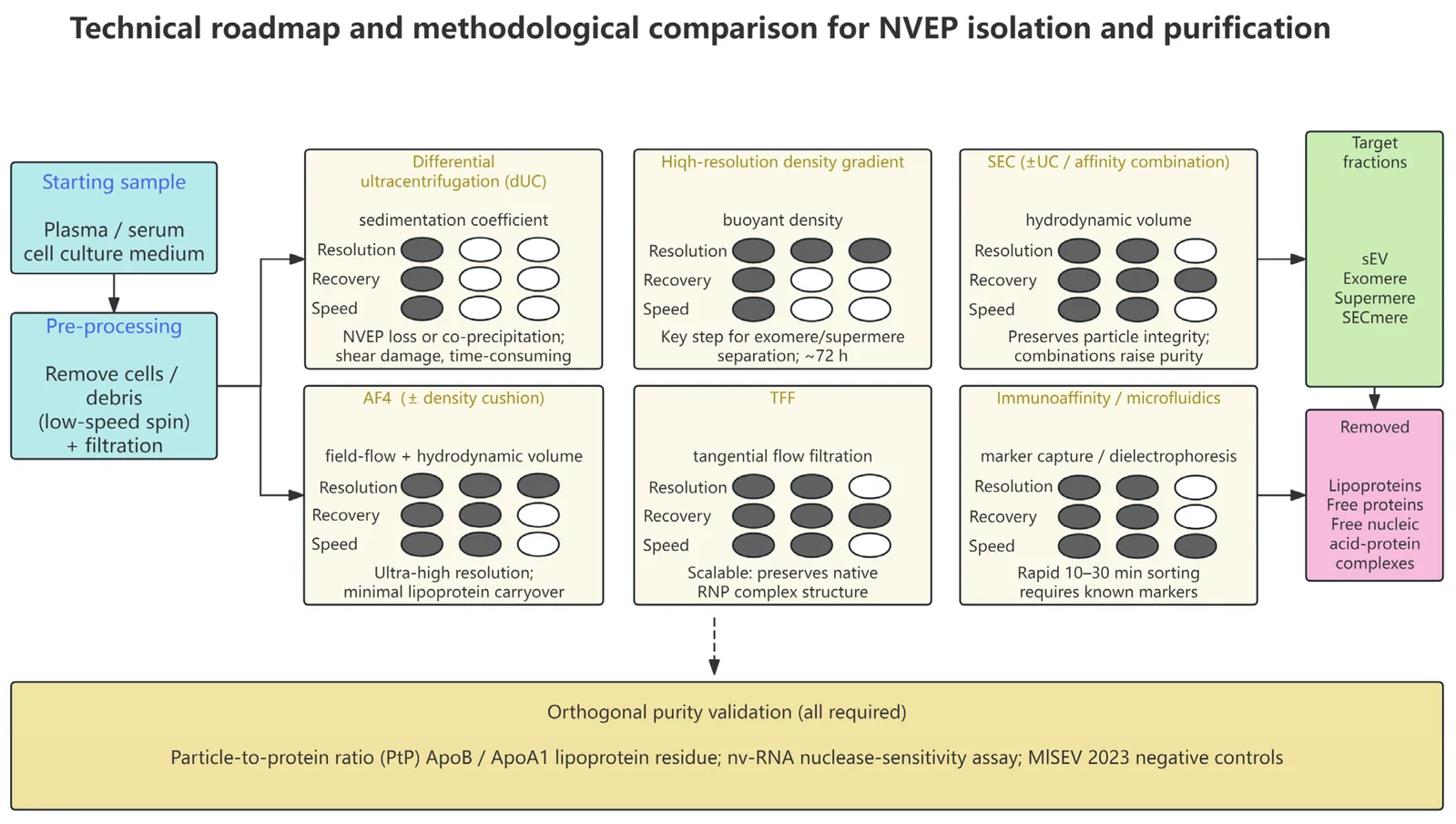
Technical roadmap and methodological comparison for NVEP isolation and purification. Dots in each method card indicate relative levels of resolution, recovery and speed (three dots = high); the bottom band summarizes the mandatory components of orthogonal purity validation.

To overcome the limitations of conventional centrifugation, various high-resolution fractionation techniques have been introduced into the NVEP isolation field, each with its own strengths and weaknesses. Size-exclusion chromatography (SEC) is widely used because of its operational simplicity, low cost, and good preservation of particle integrity. However, when used alone, it still suffers from residual lipoproteins and free proteins, and the resulting purity is often lower than that achieved by ultracentrifugation [43,47]. To improve its performance, researchers have developed two-step protocols combining SEC with ultracentrifugation, or coupled SEC with affinity capture techniques such as SmartSEC, which significantly improve the purity of plasma-derived NVEPs [45,47]. Asymmetric flow field-flow fractionation (AF4) offers even higher resolution and enables fine fractionation of submicron particles based on their hydrodynamic volumes. When combined with density cushion ultracentrifugation, AF4 can yield highly purified EV and NVEP fractions with extremely low lipoprotein contamination from plasma, leading to the successful identification of more than 1,000 associated proteins [31,48]. Density gradient centrifugation can effectively distinguish particle subpopulations of different densities and is a critical step for isolating exomeres and supermeres. Nevertheless, it is laborious, requires up to 72 h, and yields relatively low recovery rates [14,32]. Emerging integrated isolation protocols attempt to combine differential centrifugation, filtration, concentration, and high-resolution density gradient fractionation, enabling the sequential isolation of large and small EVs, exomeres, and supermeres from the same starting material with ensured purity and reproducibility [14]. In addition, super-absorbent polymers (SAPs) combined with SEC have proven to be an effective alternative to ultracentrifugation, improving purity while maintaining relatively high yields [46]. The overall technical workflow for NVEP isolation and purification, together with the respective roles of each method, is illustrated in Figure 2. A comprehensive comparison of these techniques in terms of resolution, purity, recovery, and throughput is presented in Table 3. Beyond these approaches, immunoaffinity capture reagents [49], microfluidic platforms [50,51], hybrid tangential flow filtration systems [52], and isoelectric focusing fractionation [53] have also been adapted for the isolation of NVEPs. Technical choices should be optimized according to the sample type, the downstream application, and the trade-off between purity and yield, as a single technique can rarely meet the stringent requirements of NVEP research.

### 3.3. Potential and Limitations of Immunoaffinity Capture and Microfluidic Techniques in Subpopulation Isolation

Immunoaffinity capture and microfluidic techniques offer new solutions for the precise isolation of specific NVEP subpopulations. Immunoaffinity methods exploit unique molecular markers on the NVEP surface for specific enrichment. For example, a method based on glycosaminoglycan (GAG)-functionalized magnetic beads (LipoMin) removes 98.2% of ApoB contamination within 10 min while retaining 93% of CD81-positive particles, markedly outperforming conventional density gradient methods [32]. The SHINER workflow, through the SWITCHER tool, enables the gentle recovery and multi-marker adaptation of specific exosome/NVEP subpopulations while preserving particle structural integrity and biological function [49]. However, such techniques rely heavily on known and specific surface markers. Given that specific markers for NVEPs remain poorly defined, their broad application is currently limited [11]. Microfluidic techniques, in contrast, display unique advantages in processing small-volume and precious samples, featuring rapid operation, high throughput, automation, and low sample consumption [54,55,56]. For example, a microfluidic device based on electrothermal vortices and dielectrophoretic forces achieves label-free, continuous-flow size sorting in high-conductivity media, with both recovery and purity reaching approximately 80% [50]. The ExoCPR platform integrates bubble-driven micromixing with immiscible filtration and completes the high-purity isolation of plasma NVEPs within 29 min [51]. Nevertheless, the complex design, manufacturing cost, and insufficient standardization of microfluidic chips remain bottlenecks for their large-scale adoption, and some active separation methods, such as acoustic sorting, provide high purity but relatively low yields [43]. Future efforts should focus on developing affinity reagents targeting NVEP-specific molecules and promoting the standardization and good manufacturing practice (GMP)-grade production of microfluidic platforms to meet the requirements of clinical translation [33,57].

### 3.4. Current Status of Purity Assessment: How to Exclude Contamination by Lipoproteins and Free Nucleic Acid–Protein Complexes

The core challenge in NVEP isolation lies in the substantial overlap in physicochemical properties between NVEPs and lipoproteins, particularly high-density lipoprotein (HDL) and low-density lipoprotein (LDL), as well as free nucleic acid–protein complexes. This overlap makes purity assessment a critical step in methodological validation. Conventional total protein quantification or single-marker Western blotting is no longer sufficient for comprehensively evaluating NVEP purity. Multidimensional orthogonal validation strategies are therefore required. Proteomic analyses have shown that even with optimized AF4 or SEC isolation, interference from residual high-abundance plasma proteins remains a concern. The particle-to-protein (PtP) ratio has been proposed as a quantitative indicator, although PtP values may be inconsistent with tetraspanin quantification obtained by high-resolution nano-flow cytometry (nFCM) [44]. For lipoprotein contamination, the specific detection of ApoB and ApoA1 is essential. For example, LipoMin treatment achieves an ApoB removal rate of 98.2% and reduces ApoA1 by 76.5% [32], and the ExoTFF system reduces impurity levels by 80% with an 800% increase in the purity ratio [52]. To discriminate free nucleic acid–protein complexes, nuclease sensitivity assays should be combined with high-resolution imaging techniques, because the nv-RNA within NVEPs exhibits stability features distinct from those of free RNA [17]. In addition, the MISEV guidelines emphasize that negative control data for non-EV components, such as lipoproteins and ribonucleoproteins, should be reported to confirm that the obtained signals genuinely originate from NVEPs rather than co-purified contaminants [33,58]. As no absolute gold-standard reference material is currently available, establishing comprehensive quality control indicators covering multiple potential contaminants is a necessary step toward improving the reproducibility of NVEP research [8].

### 3.5. Automated Isolation Platforms: Lessons from the EV Field and the Unmet Need for NVEP-Adapted Systems

In the EV field, isolation has rapidly evolved from manual, operator-dependent protocols toward automated and high-throughput platforms that improve standardization and reproducibility. EXODUS couples negative-pressure oscillation with double-coupled harmonic oscillator-induced membrane vibration to achieve automated, label-free purification of exosomes from diverse biofluids, outperforming conventional techniques in speed, purity, and yield, and has been validated at the cohort scale on urine samples from 113 patients [59]. Automated size-exclusion chromatography workstations (e.g., the ExoFaster series) enable standardized, walk-away processing of multiple samples in parallel with preset protocols and automated cleaning procedures, and the recently reported ExoFAST microfluidic platform implements automated pinched-flow fractionation, achieving particle recovery comparable to ultracentrifugation with reproducible, operator-independent processing [60]. However, these platforms were designed and calibrated for vesicular particles of approximately 40–150 nm. Their membrane cutoffs, fractionation windows, and flow parameters have never been optimized or validated for membrane-free particles smaller than 50 nm. Whether NVEPs are efficiently recovered by such systems, or instead pass through the separation membranes together with free proteins and ribonucleoprotein complexes, remains unknown. The development of NVEP-adapted automated platforms—such as AF4-or isoelectric-focusing-based modules with sub-50 nm fractionation windows coupled with inline purity monitoring—should therefore be regarded as a methodological priority for the maturation of NVEP research.

## 4. Functional Mechanisms of NVEP Cargo and the Reassignment of Exosome Functions

**Figure 3:**
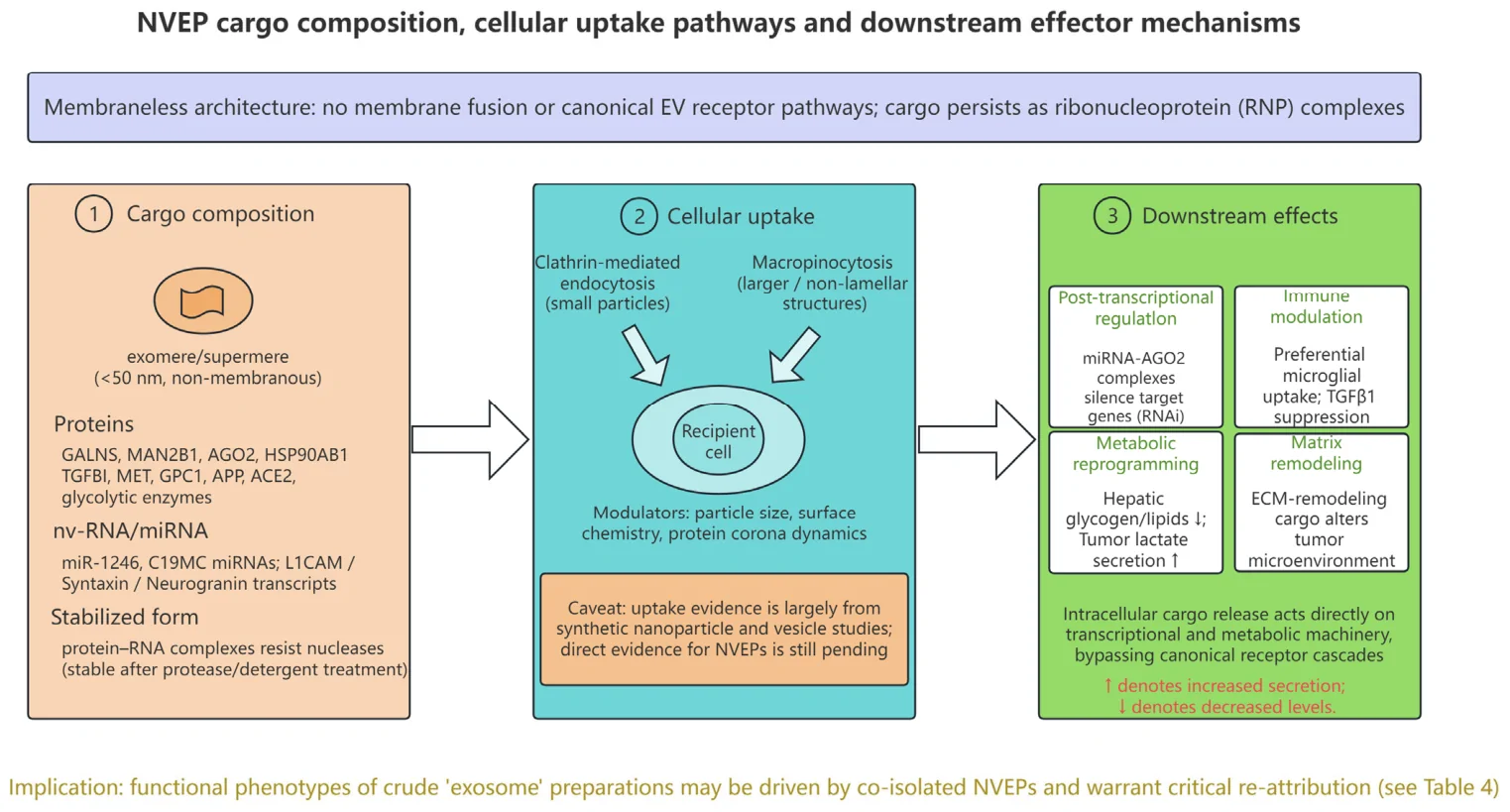
Framework of NVEP cargo composition, cellular uptake pathways and downstream effector mechanisms. ① Cargo persists as nuclease-resistant protein–RNA complexes; ② uptake pathways differ from canonical EV routes, though direct evidence for NVEPs remains limited; ③ downstream effects span post-transcriptional regulation, immune modulation, metabolic reprogramming and matrix remodeling.

This section systematically describes the unique biological behaviors of NVEPs that distinguish them from conventional vesicles and critically re-evaluates the functional properties previously attributed to exosomes. With the discovery of membrane-free nanoparticles such as exomeres and supermeres, studies have confirmed that proteins and nucleic acids are enriched in these particles to a greater extent than was previously recognized for exosomes alone. This finding necessitates a reassessment of the functional attribution of extracellular communication at three levels: molecular stability, uptake pathways, and downstream effects [11,12]. NVEPs differ from lipid bilayer-enclosed vesicles not only in their physicochemical properties. Their existing forms in biological fluids, their mechanisms of entry into recipient cells, and the biological effects they mediate all display distinct specificity, suggesting that many phenomena previously attributed collectively to “exosome functions” may need to be reassigned to NVEP subpopulations [14,28]. The overall framework of the cargo composition, cellular uptake pathways, and downstream effector mechanisms of NVEPs is illustrated in Figure 3.

### 4.1. Mechanisms Underlying the Extracellular Stability of nv-RNA: From “Degradation Products” to Functional Carriers

For a long time, nv-RNA that is not enclosed by a lipid bilayer has been regarded as a product of cell lysis or metabolic waste because of its sensitivity to ribonucleases. However, recent studies have shown that, despite the lack of membrane protection, some nv-RNA displays relative stability in biological fluids and tissue culture media. It also performs unique biological functions centered on intercellular communication rather than protein synthesis [17]. The maintenance of this stability may rely on the complex structures formed between nv-RNA and specific proteins, which enable it to resist enzymatic degradation in the extracellular environment. For example, in AD research, SECmeres smaller than 50 nm in diameter have been identified in the human brain and blood. Although these particles lack the characteristics of sEVs, they are enriched in RNAs associated with genes such as L1CAM, Syntaxin, and Neurogranin. Moreover, these RNAs showed higher significance than sEV-derived RNAs in distinguishing patients from controls [16]. This finding suggests that nv-RNA may not be randomly degraded fragments but rather selectively packaged functional carriers with pathophysiological significance. However, this conclusion is currently derived mainly from a single cohort study and requires independent validation. In addition, exomeres and supermeres have been found to be enriched in various bioactive molecules, particularly RNA species once mistakenly believed to be delivered exclusively by exosomes, further supporting the possibility that NVEPs serve as independent RNA carriers [11]. Therefore, nv-RNA should be redefined as a functional informational molecule that achieves extracellular stability through membrane-independent mechanisms. Understanding its stabilization mechanisms is a prerequisite for elucidating the biological functions of NVEPs.

### 4.2. Cellular Uptake and Receptor Recognition: Clathrin-Mediated Endocytosis, Macropinocytosis, and Other Pathways Distinct from Those of Classical EVs

Classical EVs are internalized by recipient cells through a broad repertoire of routes, including clathrin- and caveolin-mediated endocytosis, macropinocytosis, phagocytosis, lipid raft-mediated internalization, direct membrane fusion, and receptor-mediated recognition such as phosphatidylserine (PS)-dependent uptake [61,62]. Because NVEPs lack a lipid bilayer, they cannot exploit membrane fusion and are unlikely to engage PS-dependent recognition; their uptake is therefore expected to rely predominantly on endocytic pathways and to display marked heterogeneity and size dependence. In the absence of direct experimental evidence for NVEPs, the following discussion attempts to infer their possible entry routes from studies on synthetic nanoparticles and vesicular particles of comparable size and surface properties; these inferences are necessarily indirect and must be validated in bona fide NVEP preparations. Studies have shown that the cellular uptake patterns of nanoparticles are highly dependent on their physicochemical properties and the recipient cell type, involving endocytosis, phagocytosis, and other active transport mechanisms [63]. For smaller NVEPs or analogous nanostructures, clathrin-mediated endocytosis (CME) has been confirmed as a key entry route. For example, PIMA-PEG-coated upconversion nanoparticles are internalized by tumor cells mainly through CME, with a preference for this pathway at longer time points [64]. Similarly, the uptake of DNA scaffold-based poly (lactic-co-glycolic acid) (PLGA) nanoparticle systems has been confirmed to be energy-dependent and to follow the CME pathway [65]. For larger particles or those with non-lamellar structures, in contrast, macropinocytosis predominates. Studies have found that non-lamellar lipid nanoparticles, particularly those in the cubic phase, achieve significantly enhanced cellular uptake mainly through active endocytic pathways dominated by macropinocytosis [66]. Plasma membrane-derived microvesicles with diameters ranging from 100 nm to 1 μm can also be internalized through macropinocytosis involving lamellipodia formation [67]. In addition, the uptake of NVEPs is dynamically regulated by surface modifications and the protein corona. For instance, PEGylation accelerates the uptake of amino-modified silica nanoparticles, whereas pre-coating with bovine serum albumin (BSA) slows the uptake rate [68]. It should be emphasized, however, that this corona-related evidence derives from synthetic nanoparticles [68] and vesicular EVs [21,22,23,24]; direct proteomic characterization of coronas formed on exomeres or supermeres in biological fluids has not been reported and constitutes an important open question for the field. The above evidence on uptake pathways derives mainly from studies of synthetic nanoparticles and vesicles [63,64,65,66,67], and direct experimental evidence on whether NVEPs adopt the same internalization routes is still lacking. Systematically elucidating the cellular uptake mechanisms of NVEPs and their impact on intracellular fate is therefore an urgent issue to be addressed in this field.

### 4.3. Downstream Effector Mechanisms: Post-Transcriptional Regulation, Metabolic Reprogramming, and Signal Transduction

After entering recipient cells, NVEPs deliver specific protein, RNA, and metabolite cargo and trigger a series of downstream biological effects that differ from those of conventional EVs. At the level of post-transcriptional regulation, the specific RNAs carried by NVEPs can directly modulate target gene expression. For example, after entering the CNS, supermeres preferentially interact with microglia and suppress transforming growth factor β1 (TGFβ1) expression, thereby modulating immune activity [28]. In the reproductive system, placental trophoblast-derived exomeres have been shown to carry C19MC miRNAs, suggesting a potential regulatory role in maternal–fetal interface communication. Regarding metabolic regulation, current evidence derives mainly from supermeres. A single-dose in vivo experiment showed that cancer-derived supermeres reduce hepatic glycogen and lipid levels [15], suggesting that NVEPs may participate in systemic metabolic regulation. However, this evidence is insufficient to establish a general function of NVEPs as metabolic regulators. In addition, NVEPs participate in ECM remodeling. Supermeres are enriched in relevant components, enabling them to influence the physical properties of the tumor microenvironment (TME) [28]. More importantly, NVEP functions often do not depend on classical membrane receptor signaling cascades. Instead, they may act directly on the transcriptional machinery or metabolic enzyme systems through the intracellular release of cargo. For example, the non-vesicular dysfunctional mitochondria released by melanoma cells, although not falling within the typical definition of NVEPs, demonstrate that non-vesicular organelle release serves as an alternative quality control mechanism. This process can alter the extracellular microenvironment and thereby influence tumor burden and prognosis [39]. The above findings suggest that the functional execution of NVEPs may be independent of the vesicular system, although the connections among these mechanisms remain unclear.

### 4.4. Critical Re-Evaluation of Previously Assigned Exosome Functions

With the development of high-resolution separation techniques, a large body of bioactive substances and functions previously attributed to exosomes may in fact originate from NVEPs. This situation requires a critical re-evaluation of the existing literature. First, proteomic and RNA sequencing data show that proteins and nucleic acids are enriched in exomeres and supermeres to a greater extent than was previously recognized for exosomes alone. This implies that the functional phenotypes obtained with crude “exosome” preparations in the past were likely driven mainly by co-isolated NVEPs [11,12]. Second, in the field of disease biomarkers, the RNAs carried by SECmeres outperform those of sEVs in distinguishing patients with AD and are enriched in brain endothelial cell transcripts. This suggests that the marker signals previously attributed to “brain-derived exosomes” may actually originate from this non-vesicular subpopulation [16]. Third, regarding cross-barrier delivery, supermeres have been shown to cross the BBB in a manner dependent on their intact structure and RNA content and to selectively accumulate in glioblastoma (GBM) tumors in vivo [28]. This property was previously often attributed collectively to the natural homing ability of exosomes. In addition, the marked differences in lipid, protein, and RNA composition between NVEPs and EVs in CRC and GBM endow them with distinct functional properties [28]. Representative cases of functional reassignment reported to date are summarized in Table 4. Therefore, in future studies involving the functional dissection of extracellular particles smaller than 100 nm, interference from NVEPs must be excluded through rigorous approaches such as high-resolution density gradient fractionation. Alternatively, the functions should be explicitly reassigned to NVEPs to correct the cognitive bias in this field [14].

### 4.5. Synergy and Antagonism Between NVEPs and Vesicular EVs: An Integrated Regulatory Model

NVEPs and vesicular EVs do not operate in isolation. Instead, they constitute a dynamically interacting and integrated regulatory network in the extracellular space. The two may act synergistically to maintain tissue homeostasis or promote disease progression. For example, in the TME, EVs can promote angiogenesis and fibroblast transformation [69], whereas NVEPs such as supermeres are responsible for matrix remodeling and immune regulation [28]. The two may therefore coordinate in space and time to construct a pro-tumorigenic niche. Conversely, NVEPs and EVs may also engage in competitive antagonism or mutual regulation. A “nanomaterial-EV regulatory axis” has been proposed, in which the physicochemical properties of nanomaterials, including synthetic and endogenous non-vesicular particles, can modulate EV release, cargo enrichment, and downstream processes. In turn, EVs can internalize nanoparticles and trigger oxidative stress or inflammation [70]. This implies that the presence of NVEPs may feedback-regulate the cellular secretion of EVs, or that the two may compete for the same cellular uptake receptors, resulting in functional antagonism. In addition, cell density has been shown to influence the diffusion and internalization of EVs. Densely packed cells promote the internalization and degradation of EVs by adjacent cells and limit their diffusion [20]. This local microenvironmental effect may also apply to NVEPs, leading to complex concentration-dependent interactions between the two in short-range communication. Constructing an integrated regulatory model that encompasses both NVEPs and EVs requires comprehensive consideration of their production ratios, clearance kinetics, and differential responses of the same target cells. This is a critical step toward a full understanding of the intercellular communication system.

## 5. Evidence of NVEPs in Disease Contexts

### 5.1. Cancer: Multi-Omics Evidence in Colorectal Cancer (Cargo Differences Between 2D and 3D Cultures) and Drug Resistance Transfer

In CRC research, NVEPs, particularly supermeres, have been confirmed to carry unique functional cargo and mediate key malignant phenotypes. Studies have shown that cancer-derived supermeres are enriched in glycolytic enzymes, TGFBI, miR-1246, MET, GPC1, and AGO2, a cargo profile that differs significantly from that of conventional exosomes [15]. At the functional level, CRC-derived supermeres can increase lactate secretion and reshape the tumor metabolic microenvironment. They have also been confirmed to transfer resistance to cetuximab, suggesting that NVEPs play a key role in tumor therapeutic resistance that is independent of vesicular EVs [15]. In addition, the dimensionality of the culture system significantly influences the molecular profiles of NVEPs. Comparative studies have found that, regardless of the isolation method used, exomeres and supermeres obtained from 3D hollow-fiber bioreactor cultures consistently differ in their proteomic features from those obtained under 2D culture conditions. Meanwhile, small RNAs and their base modifications, as well as lipidomic profiles, are jointly influenced by culture conditions and purification strategies [29]. At the molecular marker level, GALNS and MAN2B1 have been reported as specific molecular markers of exomeres, and supermeres are likewise enriched in cancer-associated cargo such as MET and GPC1 [15,30]. The above multi-omics evidence indicates that NVEPs possess specific molecular features in CRC that are distinct from those of vesicular EVs [29].

### 5.2. Nervous System: The Blood–Brain Barrier Crossing Behavior of Supermeres and Their Association with AD

The mechanisms of NVEPs in CNS diseases are gradually being disentangled from conventional exosome research. Owing to their intact structure and specific RNA content, supermeres have been confirmed to be capable of crossing the BBB. After entering the CNS, they preferentially interact with microglia and suppress TGFβ1 expression, thereby regulating microglial immune activity [28]. In the pathological context of AD, supermeres have been found to be enriched in amyloid precursor protein (APP). The identification of this key cargo suggests that NVEPs may directly participate in the pathological progression of AD rather than acting as mere bystanders [15]. In addition, the newly identified SECmere subpopulation, a class of particles smaller than 50 nm that lack typical EV features, has been found in the human brain and blood. These particles are enriched in brain endothelial cell transcripts and participate in the constitution of the BBB. Their carried RNAs show higher discriminatory power than sEV RNAs in distinguishing patients with AD from healthy controls, with specific enrichment in synapse-related molecules such as L1CAM, Syntaxin, and Neurogranin [16]. This evidence suggests that NVEPs may serve not only as carriers of pathological signals in neurodegenerative diseases but also as participants in intercellular communication across the BBB. However, their actual contribution to human disease progression remains to be validated in vivo [9,28].

### 5.3. Metabolic Organs: Regulation of Hepatic Glycogen and Lipid Metabolism by Supermeres

Preliminary evidence suggests that the effects of NVEPs, particularly supermeres, may not be restricted to the local microenvironment but may extend to distal organs. Studies have clearly shown that cancer cell-derived supermeres significantly reduce hepatic lipid and glycogen levels after in vivo administration. This indicates that NVEPs can act as effector molecules of the tumor–liver metabolic axis and mediate tumor-induced systemic metabolic reprogramming [15]. This metabolic regulatory function may be related to the highly abundant specific cargo of supermeres, whose in vivo uptake rate is markedly higher than that of sEVs and exomeres [15]. However, whether such uptake efficiency can be translated into stronger functional output remains to be verified. Current direct research on hepatic metabolism has focused mainly on the pathological effects of tumor-derived supermeres. It should be noted that studies on plant-derived nanovesicles and exosome-based therapy for non-alcoholic fatty liver disease (NAFLD) were performed with bulk vesicle preparations obtained by conventional isolation protocols, which are now recognized to co-isolate NVEPs [71,72]. The reported effects can therefore neither be attributed to vesicular particles with certainty nor be directly extrapolated to NVEPs, and whether NVEPs contribute to these therapeutic outcomes remains to be tested. Direct evidence on whether NVEPs have the potential to intervene in liver diseases is currently lacking. Nevertheless, it must be emphasized that the evidence confirming the direct regulation of hepatic glycogen and lipid metabolism by supermeres currently derives mainly from the study in [15]. This provides a novel non-vesicular mechanistic explanation for understanding cancer cachexia and distal organ metabolic disorders.

### 5.4. Reproductive System: Research Gaps and Prospects for Placenta-Derived Exomeres

**Figure 4:**
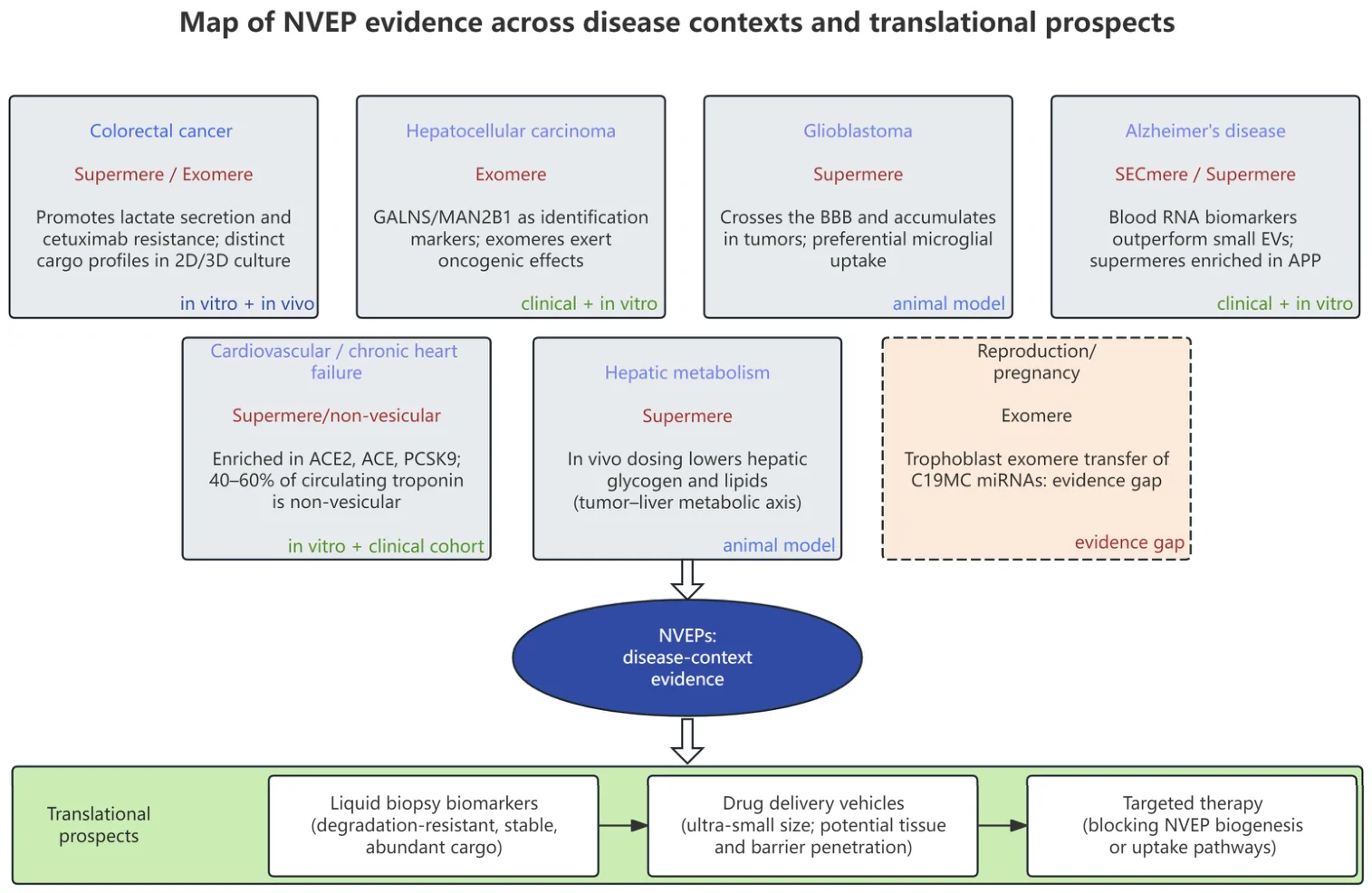
Map of NVEP evidence across disease contexts and translational prospects. Each node indicates the particle type, key findings and evidence strength; the reproduction/pregnancy field currently lacks direct evidence (dashed box).

Compared with the fields of cancer and neuroscience, research on NVEPs in the reproductive system is still at a very early stage, and the available literature is extremely limited. A literature search revealed no direct empirical report on the carriage of C19MC miRNAs by placental trophoblast-derived exomeres. Although integrated protocols for the sequential isolation of exomeres and supermeres from cell culture media and human plasma have been established and their applicability across multiple tissues has been confirmed [14], and although the Vesiclepedia database has incorporated data on non-vesicular particles, including supermeres and exomeres [41], functional validation of the exomere-mediated transport of trophoblast-specific C19MC miRNAs in the BeWo cell line or in placental trophoblasts has likewise not been reported. Therefore, following strict evidence-based principles, this section cannot provide a detailed discussion based on the existing literature. This situation precisely reflects the research gap and urgent demand in the field of NVEP reproductive biology. Targeted studies are urgently needed to verify the potential roles of exomeres in maternal–fetal interface communication and pregnancy-related diseases. Integrating the evidence from Section 5.1, Section 5.2, Section 5.3 and Section 5.4, the empirical NVEP findings in each disease field and their evidence strength are summarized in Table 5. The correspondence among NVEP particle types, key findings, and translational directions in each disease field is illustrated in Figure 4.

## 6. Translational Prospects: Biomarkers and Therapeutic Potential

### 6.1. Mining NVEP Cargo as Liquid Biopsy Biomarkers: Advantages of High Abundance, Strong Stability, and Absence of Membrane Degradation

The potential of NVEPs as liquid biopsy biomarkers is gradually being recognized. Their core advantage lies in the exceptional stability and detection sensitivity conferred on cargo molecules by their unique physicochemical properties. Unlike conventional EVs, NVEPs such as exomeres and supermeres lack a lipid bilayer. This feature protects the encapsulated bioactive molecules from membrane-degrading enzymes, allowing them to maintain greater integrity in circulating body fluids [11]. Studies have shown that exomere-encapsulated miRNAs remain strongly resistant to nuclease degradation even after protease and surfactant treatment. This confirms their nature as highly stable non-vesicular complexes and suggests that NVEP cargo may possess favorable stability in circulation, providing advantageous conditions for their use as biomarkers. However, direct measurements of their in vivo half-life are still lacking [18]. In addition, NVEPs are enriched in various proteins and RNAs previously attributed to exosomes, and their enrichment in certain fractions even exceeds that of conventional EVs. For example, supermeres are highly enriched in argonaute proteins (AGO1 and AGO2), which are undetectable in high-resolution density gradient-purified sEVs, and they constitute a greater source of extracellular RNA than either sEVs or exomeres; they are additionally replete with glycolytic enzymes (e.g., ENO2 and LDHA) and disease-associated proteins such as TGFBI [12,15]. This means that more abundant disease-associated signals may be obtained from the same sample volume. Although the current liquid biopsy field focuses mainly on components such as EVs, circulating tumor DNA (ctDNA), and cells [73,74], NVEPs are expected to compensate for the deficiencies of existing biomarkers in stability and abundance, owing to the degradation resistance conferred by their membrane-free structure and their specific cargo profiles. In particular, in clinical scenarios requiring long-term monitoring or involving restricted sample processing conditions, the stability of NVEP cargo makes it a highly promising complementary or alternative biomarker source. Nevertheless, clinical translation still requires overcoming the challenges of isolation purity and standardized detection, ensuring that the measured signals genuinely originate from NVEPs rather than residual EVs or free protein complexes [14,75].

### 6.2. NVEPs as Drug Delivery Carriers: Proof of Concept and Prospects for Design Principles

Although direct research on NVEPs as drug delivery carriers is still in its infancy, their unique application potential and design strategies can be envisioned based on their biological properties and general principles in nanomedicine. Existing drug delivery research emphasizes that the particle size, surface chemistry, and shape of nanoparticles are key determinants of their penetration behavior, targeting efficiency, and in vivo distribution [76,77]. NVEPs naturally possess ultra-small particle sizes and a membrane-free structure. Theoretically, they may therefore exhibit stronger tissue penetration than conventional liposomes or EVs and may display natural advantages similar to those of supermeres in crossing physiological barriers such as the BBB [78]. Regarding design principles, tangential flow filtration (TFF) has been proven suitable for the scalable isolation of high-purity exomeres, and the resulting products retain their native RNAi protein complex structures. This lays a process foundation for developing NVEP-based nucleic acid drug delivery platforms [18]. Drawing on the experience of synthetic nanocarriers, the design of future NVEP carriers may need to incorporate surface engineering modifications to enhance targeting or modulate immunogenicity, for example, through functionalization with nanobodies or small-molecule ligands [79]. At the same time, the potential immune risks of non-vesicular particles must be carefully considered. Animal experiments have shown that non-PEGylated nanoparticles can induce the accelerated blood clearance (ABC) phenomenon, which is associated with enhanced neutrophil phagocytic activity. This suggests that the immune safety of repeated administration must be strictly evaluated when developing NVEP-based therapies [80]. In addition, given that NVEPs lack a lipid membrane, conventional membrane fusion-based loading strategies may not be applicable. Novel loading mechanisms based on protein–RNA interactions or electrostatic adsorption need to be explored. Alternatively, hybrid strategies, such as hybridization with lipid nanoparticles (LNPs), could be used to integrate the functionality of NVEPs with the loading capacity of synthetic carriers [81,82].

### 6.3. Therapeutic Strategies Targeting NVEP Biogenesis or Uptake Pathways

Targeting the biogenesis or uptake pathways of NVEPs is a potential new strategy for intervening in intercellular communication, but this field still faces the challenge of insufficient mechanistic understanding. The existing literature indicates that the biogenesis of NVEPs, such as exomeres and supermeres, remains an unsolved mystery with only indirect clues. This is in sharp contrast to the relatively well-defined ESCRT-dependent and ESCRT-independent pathways of EVs [11,12]. Nevertheless, given that NVEPs carry large amounts of functional proteins and RNAs and participate in disease progression, blocking their specific secretion or uptake may become a new therapeutic strategy. Regarding uptake mechanisms, although the specific receptors have not been fully identified, the endocytic behavior of nanoparticles is known to be regulated by particle size and surface properties. Pathways distinct from those of classical EVs may involve CME or macropinocytosis [76,83]. Any such blockade strategy remains speculative at present: it is contingent on the prior identification of NVEP-specific surface markers or receptors and on direct evidence that NVEPs, rather than co-isolated vesicles, transmit the pathological signals in question. We therefore present this concept strictly as a long-term perspective rather than an immediately actionable strategy. If NVEPs are confirmed to play a key role in this process, targeting their communication links would help break this vicious cycle. In addition, physicochemical methods such as saponin treatment have been shown to differentially extract the contents of biological nanoparticles, implying that different particle types possess different structural stabilities [84]. This provides a potential methodological avenue for selectively disrupting NVEPs without affecting the function of normal EVs. Future therapeutic development urgently requires the integration of high-resolution spatiotemporal mapping and multi-omics technologies. Only by first identifying the specific biogenesis regulators and cell surface recognition molecules of NVEPs under particular pathological states can precise targeting be achieved [14,85].

## 7. Theoretical Controversies, Research Limitations, and Unresolved Questions

### 7.1. Ambiguity of the “Non-Vesicular” Definition and Nomenclature Disputes

Although the discovery of NVEPs such as exomeres and supermeres has revealed the complexity of intercellular communication, their definition and nomenclature still face significant challenges [12,27]. The current classification system relies mainly on operational definitions. NVEPs are defined through specific isolation procedures, such as the re-fractionation of the supernatant after ultracentrifugation, rather than by a clear biogenesis mechanism or unique molecular markers [14]. This isolation-based definition renders the boundary of the term “non-vesicular” inherently ambiguous. Differences in the separation parameters used by different laboratories may lead to heterogeneity in the obtained fractions and thereby undermine the uniformity of nomenclature [25,86]. In addition, with the discovery of new subtypes such as SECmeres, the internal heterogeneity of NVEPs has further increased. These particles differ from conventional sEVs and from known exomeres and supermeres in both size (<50 nm) and cargo composition, making the existing binary classification of vesicular versus non-vesicular appear overly simplistic [16]. The MISEV guidelines issued by the International Society for Extracellular Vesicles (ISEV) attempt to standardize the distinction between EVs and non-vesicular particles [33]. However, they still emphasize a multidimensional operational definition that integrates the source material, isolation method, and molecular features. This reflects that no single physical or biological criterion is currently sufficient to precisely delineate the scope of NVEPs [25]. Moreover, certain bioactive molecules, such as Argonaute proteins, can exist in both vesicular and non-vesicular forms, and the two forms differ in protease resistance and immunogenicity. This further aggravates the limitations of classification based solely on physical morphology [87]. Therefore, establishing a universal nomenclature and classification standard that is independent of specific isolation techniques and reflects the essential biological attributes of NVEPs is an urgent theoretical issue in this field.

### 7.2. Functional Differences Between In Vitro Culture Systems and the In Vivo Physiological Environment

The vast majority of current functional studies on NVEPs are based on in vitro cell culture systems. These systems differ substantially from the complex in vivo physiological environment, which limits the translational value of the conclusions. Studies have shown that culture conditions and purification strategies significantly influence the cargo composition of extracellular particles. Even under standardized operations, the proteomic profiles of exomeres and supermeres produced in vitro may not fully represent their true state in situ in tissues or body fluids [29]. For example, in the TME, the functions of NVEPs are dynamically regulated by multiple factors, including matrix stiffness, immune cell infiltration, and metabolic gradients. These factors are difficult to simulate accurately in 2D or conventional 3D cultures [28]. In addition, the uptake efficiency and downstream signal activation of NVEPs observed in vitro are often obtained under non-physiological conditions of high concentration and short exposure, which may exaggerate their biological effects. In contrast, NVEPs in the in vivo circulation face severe challenges such as nuclease degradation, immune clearance, and blood flow shear forces. Their half-life, targeting ability, and actual delivery efficiency may differ considerably from in vitro results [18,28]. In neuroscience research in particular, although supermeres have been confirmed to cross the BBB and accumulate in GBM, this phenomenon was validated in specific animal models. Its conservation and functional weight in human pathophysiological processes still require cautious evaluation [28]. Therefore, the lack of research models that faithfully simulate the in vivo microenvironment, together with the systematic absence of in vivo validation for in vitro findings, constitutes a major bottleneck in current functional characterization.

### 7.3. Constraints of the Absence of Gold-Standard Reference Materials on Quantitative Research

The quantification of NVEP research is severely hindered by the absence of gold-standard reference materials, which makes cross-laboratory data comparison and quality control extremely difficult. Unlike synthetic nanoparticles or certain well-established viral vectors, NVEPs exhibit high heterogeneity and instability. No internationally recognized, rigorously characterized NVEP standards are currently available for calibrating isolation efficiency, purity assessment, or content determination [25,88]. Existing measurement techniques have limitations in both sensitivity and specificity. For example, nanoparticle tracking analysis (NTA) has limited capability for detecting particles smaller than 50 nm, whereas MS can provide protein fingerprints but cannot be directly converted into particle numbers [89,90]. Owing to the lack of a unified quantitative benchmark, the reported yields, RNA loading, and functional potency of NVEPs often differ by several orders of magnitude across studies, casting doubt on the reproducibility of the literature [8,25]. Although attempts have been made to develop multiparametric single-particle characterization methods such as SEVEN-UP, or to improve measurement accuracy using microstructured optical fiber-assisted NTA, these techniques have not yet been adopted as standardized quality control tools [90,91]. In addition, regulatory classification and measurement standards for such novel biological nanoparticles remain undefined, further impeding their clinical approval pathways as diagnostic markers or therapeutic agents [25,92]. Therefore, developing NVEP reference materials with defined composition, stable properties, and traceability, together with supporting standardized quantitative detection methods, is a key prerequisite for advancing this field from qualitative description to precise measurement.

### 7.4. Obstruction of Mechanistic Analysis by the Absence of Databases and Annotation Systems

Compared with the relatively well-established database resources in the EV field, the extreme scarcity of NVEP-specific databases and functional annotation systems severely restricts the in-depth dissection of their molecular mechanisms. Existing EV databases mainly contain molecular information on vesicles based on lipid bilayer structures. They lack dedicated annotation entries for the protein–RNA interaction patterns, unconventional secretion signals, and metabolite combinations that are unique to membrane-free NVEPs [8]. Consequently, when performing multi-omics data analysis, researchers can only borrow the annotation frameworks of EVs or intracellular complexes, which can easily lead to misinterpretation or omission of NVEP-specific functions. For example, the ribonucleoprotein (RNP) complexes formed by specific miRNAs enriched in NVEPs and their binding proteins such as AGO2 may have stabilization mechanisms and target gene regulatory networks entirely different from those of vesicle-encapsulated miRNAs. However, current bioinformatics tools cannot effectively distinguish between these two carrier forms [17,18]. In addition, owing to the lack of an integrated NVEP knowledge base, potential markers identified in different studies, such as L1CAM and Neurogranin, are difficult to cross-validate systematically or to analyze for functional associations. This hinders the transition from isolated molecular discoveries to the construction of regulatory networks [16,28]. Meanwhile, the methodological inconsistencies prevalent in EV-RNA research, such as biases in extraction reagents and differences in library preparation strategies, also exist in the NVEP field and may be even more pronounced. Without standardized data submission specifications and annotation standards, the massive omics data accumulated in the future will be difficult to integrate for effective meta-analysis [8]. Therefore, constructing multi-omics databases dedicated to NVEPs and developing bioinformatics pipelines adapted to their non-vesicular characteristics are foundational tasks for achieving in-depth mechanistic dissection of NVEPs.

## 8. Future Perspectives

NVEP research is moving from the discovery stage into a stage of mechanistic dissection and translational exploration. Future work needs to establish dedicated technical and analytical frameworks tailored to the membrane-free and small-sized physicochemical properties of NVEPs. On the one hand, their heterogeneity should be resolved at the single-particle level. On the other hand, standardized isolation and characterization procedures should be established through international collaboration to improve reproducibility across studies. The integration of spatiotemporally resolved techniques with multi-omics approaches is expected to elucidate the functions of NVEPs in physiologically relevant environments.

### 8.1. Application Prospects of Single-Particle Analysis and Integrated Multi-Omics Technologies

Given the high heterogeneity of NVEPs in size, density, and molecular composition, bulk analyses often mask the functional features of key subpopulations. Several single-particle platforms have recently become available, including corona thickness measurements by two-color STORM [93], three-dimensional single-particle tracking [94], impedance-based sub-micron fluidic analysis [95], and membrane-sensing peptide probes coupled with single-molecule arrays [96]. Among these, we consider label-free single-particle platforms the most promising for NVEPs, because they do not rely on antibody recognition of membrane-associated markers and are therefore directly compatible with membrane-free particles. Two further developments should be prioritized: single-particle multi-omics readouts, exemplified by SPIRFISH [97] and the SVAtlas resource [40], and AI-assisted classification, which already distinguishes exosomes from nanoparticles in patient plasma with very small training sets [98,99]. Rather than further incremental demonstrations on EVs, these platforms should now be adapted to sub-50 nm membrane-free particles.

### 8.2. Establishing Internationally Recognized Guidelines and Quality Control Standards for NVEP Isolation and Characterization

The most urgent priority is not any single isolation method but the establishment of NVEP-specific standards. Integrated dUC–density gradient protocols [14] and continuous isoelectric focusing [53] show that high-purity fractionation is technically feasible, and orthogonal purity panels—SEC combined with ultracentrifugation [100], styrene-maleic acid copolymer treatment for lipoprotein removal [101], particle-to-protein ratios [44], ApoB/ApoA1 residual detection [32], and nuclease-sensitivity assays [17]—are immediately implementable. We therefore argue that a MISEV-like consensus [8,25] defining the operational meaning of “non-vesicular” and the boundaries of exomeres and supermeres [27], together with validated markers such as GALNS and MAN2B1 [30] and negative controls [44,102], should take precedence over further method proliferation. Automated platforms such as 24-well SEC and digital microfluidics [102,103], and NVEP-adapted systems built on lessons from EXODUS [59] and ExoFAST [60], will deliver their full value only within such a framework.

### 8.3. High-Resolution Spatiotemporal Mapping and In Situ Functional Dissection

In vitro studies that fail to recapitulate the physiological environment can hardly reflect the dynamic behavior of NVEPs in vivo. Functional redefinition of NVEPs ultimately requires high-resolution spatiotemporal evidence obtained in physiologically relevant settings. Existing in vivo observations—supermeres crossing the BBB to preferentially interact with microglia [28] and the organ-selective maternal–fetal transfer of atmospheric magnetite nanoparticles [104]—remain fragmentary. Among the available strategies, we regard intravital imaging combined with genetically encoded tags such as Snorkel-tag [105,106] as the most promising because it enables non-destructive, subpopulation-specific tracking without relying on membrane dyes, which are incompatible with non-vesicular particles; tissue clearing and light-sheet microscopy can extend these trajectories to whole-organ, three-dimensional maps. Parallel priorities are to verify whether the entry routes inferred from synthetic nanoparticles (Section 4.2)—such as CME and macropinocytosis—operate for bona fide NVEPs [28] and to account for confounders such as 2D versus 3D culture conditions, which markedly alter the small RNA and lipid profiles of exomeres and supermeres [29]. Integration with single-cell transcriptomics [107] and spatial metabolomics will then map NVEP functions to specific cell types and anatomical locations, providing the spatiotemporal resolution needed for functional redefinition. The prioritized future technologies discussed in Section 8.1, Section 8.2 and Section 8.3 are summarized in Table 6.

## 9. Conclusions

The growing attention to non-vesicular components such as NVEPs is driving research on extracellular communication toward more refined classification and functional dissection. The discovery of NVEPs has not only filled the gap of non-membranous components smaller than 50 nm in the extracellular particle spectrum but has also prompted a revision of the long-held view that the lipid bilayer is the only carrier form enabling the stable extracellular existence of RNA. These findings suggest that the variety of mediators of intercellular information transfer may far exceed previous understanding. A key unresolved question is whether protein–RNA complexes confer functional advantages over vesicle-encapsulated cargo—for example, faster turnover or direct access to cytosolic machinery—under specific physiological and pathological conditions. From the perspective of translational medicine, the high abundance and cargo stability of NVEPs, together with the preliminarily observed tissue penetration phenomena such as BBB crossing, provide a rationale for their further investigation as liquid biopsy biomarkers and delivery carriers. However, translating this potential into clinical benefit requires acknowledging the current limitations, including ambiguous definitions, the absence of gold standards, and insufficient in vivo functional validation. Future research should integrate three dimensions that have so far been pursued separately: structural characterization of bona fide NVEPs, functional validation in vivo, and physiologically relevant experimental environments. Driven by both technological innovation and standard development, the biological identity of NVEPs can be thoroughly clarified. This endeavor is not only a complement to the EV field but also an opportunity to re-examine the material basis of intercellular communication. Addressing these challenges in a prioritized manner would convert NVEPs from an intriguing by-product of EV research into a rigorously defined and functionally validated component of intercellular communication.

## Figures and Tables

**Table 1:** Comparison of the physicochemical properties of exomeres, supermeres, SECmeres, conventional sEVs, and lipoproteins.

Particle Category	Size Range	Membrane Structure	Density/Fractionation Behavior	Representative Markers	References
sEVs (control)	30–150 nm	Present (lipid bilayer)	Buoyant density ~1.13–1.19 g/mL (sucrose); ~1.08–1.13 g/mL (iodixanol)	CD63, CD81, CD9	[12,33]
Exomeres	<50 nm	Absent	Higher-density fractions of 12-36% iodixanol gradients; pelleted only at ≥167,000× *g* for 16 h	GALNS, MAN2B1, AGO2, HSP90AB1	[13,18,30]
Supermeres	<30–50 nm	Absent	Highest-density NVEP fraction; pelleted only at ≥360,000× *g* for 16 h after exomere depletion	TGFBI, MET, GPC1, APP, ACE2	[15,29]
SECmeres	<50 nm	Absent	Resolved from sEVs by size-exclusion chromatography in a distinct elution window; buoyant density not yet quantitatively defined	L1CAM, Syntaxin, Neurogranin (RNA)	[16]
Lipoproteins (contaminant control)	HDL, 8–12 nm; LDL, 20–25 nm	Phospholipid monolayer	HDL: 1.063–1.21 g/mL; LDL: 1.019–1.063 g/mL—overlapping with the high-density NVEP zones	ApoB, ApoA1	[31,32]

**Table 2:** Summary of reported NVEP molecular markers and characteristic cargo profiles.

Particle Subtype	Markers/Characteristic Cargo	Molecular Category	Disease Association	Sample/Model	References
Exomeres	GALNS, MAN2B1	Proteins	Hepatocellular carcinoma (identification markers and tumor-promoting role)	HCC cells and patient samples	[30]
Exomeres	AGO2, HSP90AB1, and specific miRNAs	Proteins + RNA	— (RNAi carrier property)	Cell culture medium/plasma	[18]
Supermeres	Glycolytic enzymes, TGFBI, MET, GPC1, miR-1246, AGO2	Proteins + RNA	Colorectal cancer	CRC cells/animal models	[15,29]
Supermeres	APP	Protein	Alzheimer’s disease	—	[15]
Supermeres	ACE2, ACE, PCSK9	Proteins	Cardiovascular diseases	—	[15]
SECmeres	L1CAM, Syntaxin, and Neurogranin transcripts	RNA	Alzheimer’s disease (blood-based diagnosis)	Human brain/blood	[16]
Non-vesicular fraction	Troponin (40–60% of the total circulating pool)	Protein	Chronic heart failure	Patient plasma	[34]

**Table 3:** Performance comparison of NVEP isolation techniques.

Technique	Principle	Resolution/Purity	Recovery/Throughput	Time Required	Major Limitations	Representative References
Differential ultracentrifugation (dUC)	Differences in sedimentation coefficients	Low (NVEP loss or co-isolation)	Low	Long	Shear-induced damage; contamination by soluble factors	[14,42,43]
High-resolution density gradient centrifugation	Buoyant density	High (critical for separating exomeres/supermeres)	Low	Very long (approximately 72 h)	Laborious operation; low recovery	[14,32]
SEC	Hydrodynamic volume	Moderate (preserves particle integrity)	High	Short	Residual lipoproteins and free proteins	[43,47]
SEC combined with ultracentrifugation/affinity capture	Two-step protocol	High	Moderate	Moderate	Complex workflow	[45,46]
AF4 (with or without density cushion)	Hydrodynamic volume in a flow field	Very high (extremely low lipoprotein contamination)	Moderate	Moderate	Demanding instrumentation	[31,48]
Immunoaffinity magnetic beads (e.g., LipoMin)	Surface marker capture	High (98.2% ApoB removal)	High	Very short (approximately 10 min)	Dependence on known specific markers	[32,49]
Microfluidics (electrothermal flow/DEP, ExoCPR)	Size-, dielectric-, or magnetic-based enrichment	High (approximately 80% purity)	Moderate	Very short (on the order of 29 min)	Chip cost; insufficient standardization	[50,51]
TFF	Tangential membrane filtration	Moderate to high (preserves native complex structures)	High (scalable)	Moderate	Membrane fouling; requires combination with other methods	[18,52]
Isoelectric focusing fractionation	Isoelectric point	High (unbiased separation of RNPs)	High	Moderate	Emerging method with limited validation	[53]

**Table 4:** Representative cases of the reassignment of functions previously attributed to exosomes to NVEPs.

Originally Reported Function/Phenomenon	Originally Attributed Carrier	Key Evidence Supporting Reassignment	Current Attribution	References
Major carrier of extracellular RNA	Exosomes	The majority of extracellular RNA is actually associated with supermeres rather than sEVs	Supermeres	[15]
Circulating cancer-associated protein and RNA cargo: glycolytic enzymes, TGFBI, MET, GPC1, and miR-1246	Exosomes (among multiple established carriers)	These specific molecules are significantly more enriched in supermeres than in sEVs and exomeres	Supermeres (for these specific cargo molecules; EVs remain established carriers of other cancer biomarkers)	[15]
Circulating Alzheimer’s disease–associated cargo: APP	Exosomes (among multiple established carriers)	APP is enriched in supermeres	Supermeres (for APP specifically)	[15]
Circulating cardiovascular disease–associated cargo: ACE2, ACE, and PCSK9	Exosomes (among multiple established carriers)	ACE2, ACE, and PCSK9 are enriched in supermeres	Supermeres (for these specific proteins)	[15]
Blood RNA biomarkers for Alzheimer’s disease	Brain-derived exosomes	SECmere-carried RNAs show higher discriminatory power than sEV RNAs; deconvolution traced them to brain endothelial cells	SECmeres	[16]
Blood–brain barrier crossing and glioblastoma homing	Natural homing ability of exosomes	Supermeres cross the BBB depending on their intact structure and RNA content and selectively accumulate in tumors	Supermeres	[28]
Microglial immunomodulation (TGFβ1 suppression)	EVs	After entering the CNS, supermeres are preferentially taken up by microglia and suppress TGFβ1	Supermeres	[28]
Transfer of tumor drug resistance (cetuximab)	Exosomes	CRC-derived supermeres transfer drug resistance by promoting lactate secretion	Supermeres	[15]
Regulation of hepatic glycogen/lipid metabolism	Systemic metabolic factors	In vivo administration of cancer-derived supermeres significantly reduces hepatic lipid and glycogen levels	Supermeres	[15]
Extracellular miRNA-mediated RNAi	Exosomes	Exomeres are enriched in AGO2, and their miRNA cargo remains nuclease-resistant even after protease/surfactant treatment	Exomeres	[18]
Circulating troponin (heart failure monitoring)	Vesicle-associated form	In the plasma of patients with chronic heart failure, 40–60% of troponin exists in a non-vesicular form	NVEPs	[34]

**Table 5:** Summary of empirical evidence for NVEPs in disease contexts.

Disease Field	Particle Type	Key Findings	Evidence Strength	References
Colorectal cancer	Supermeres	Promote lactate secretion; transfer cetuximab resistance; enriched in glycolytic enzymes/TGFBI/MET/GPC1/miR-1246	In vitro + in vivo	[15]
Colorectal cancer	Exomeres/supermeres/EVs	Marked differences in cargo profiles among the three particle types; 2D versus 3D culture alters the proteome, small RNA modifications, and the lipidome	In vitro (multi-omics)	[29]
Hepatocellular carcinoma	Exomeres	GALNS/MAN2B1 as identification markers; exomeres exert tumor-promoting effects	Clinical samples + in vitro	[30]
Glioblastoma	Supermeres	Cross the BBB; accumulate in tumors; preferentially taken up by microglia	Animal models	[28]
Alzheimer’s disease	SECmeres	Blood RNA biomarkers show higher discriminatory power than sEV RNAs; enriched in brain endothelial transcripts	Clinical samples	[16]
Alzheimer’s disease	Supermeres	Enriched in APP cargo	In vitro	[15]
Cardiovascular diseases	Supermeres	Enriched in ACE2, ACE, and PCSK9	In vitro	[15]
Chronic heart failure	Non-vesicular fraction	40–60% of circulating troponin exists in a non-vesicular form	Clinical cohort	[34]
Hepatic metabolism	Supermeres	In vivo administration reduces hepatic glycogen and lipid levels (tumor-liver metabolic axis)	Animal models	[15]
Reproduction/pregnancy	Exomeres	Carriage of C19MC miRNAs by placental trophoblast-derived exomeres	Evidence gap; validation needed	—

**Table 6:** Prioritized future technologies for NVEP research.

Challenge	Most Promising Technology	Current Maturity	Priority Rationale	References
Isolation standardization	AF4 with sub-50 nm fractionation windows or isoelectric-focusing modules; NVEP-adapted automated platforms (lessons from EXODUS and ExoFAST)	AF4 established for NVEP discovery; automated platforms validated for EVs, NVEP-adapted versions emerging	Highest: reproducible isolation is the prerequisite for all downstream claims	[12,53,59,60]
Purity validation	Orthogonal panel: particle-to-protein ratio, ApoB/ApoA1 lipoprotein residue, nv-RNA nuclease-sensitivity assay, MISEV-style negative controls	Immediately implementable	Highest: adoptable by all laboratories without new instrumentation	[17,32,33,44]
Single-particle characterization	Label-free single-particle platforms (iSCAT, super-resolution microscopy): STORM-based corona mapping, 3D single-particle tracking, ISMA, MSP–SiMoA probes	Emerging	High: resolves subpopulation heterogeneity masked by bulk assays	[93,94,95,96]
In vivo functional tracking	Intravital imaging combined with genetically encoded tags (e.g., Snorkel-tag)	Proof of concept	High: required for functional redefinition under physiological conditions	[105,106]
Reference materials and guidelines	NVEP reference-material libraries and MISEV-like consensus guidelines	Absent	Foundational: enables cross-laboratory comparability and clinical translation	[8,25,53,100]

## Data Availability

No new data were created or analyzed in this study. Data sharing is not applicable to this article.
